# School eye health in South Asia

**Published:** 2017

**Authors:** Damodar Bachani

**Affiliations:** Deputy Commissioner (NCD), Ministry of Health & Family Welfare, Government of India & Director Professor, Department of Community Medicine, Lady Hardinge Medical College & Associated Hospitals, New Delhi, India

**Figure:**
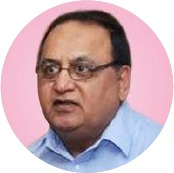
Dr Damodar Bachani

**Integrating eye health into school health programmes can provide comprehensive eye health services to millions of children all over the world.**

**Figure:**
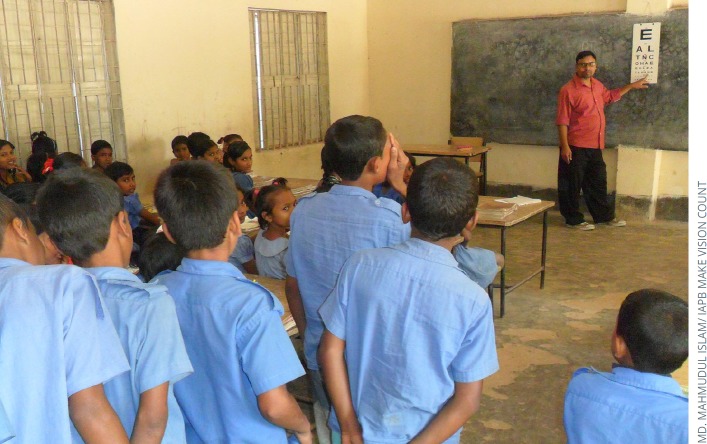
Good vision at school is essential for learning. INDIA

Primary education is a fundamental human right. It has the potential to change individuals' lives and fuel social transformation. Good health is critical for achieving a sound education and a bright future for a child. Vision is an integral part of a child's health and poor vision can have long-term impact on their social, cognitive and physical development. An estimated 1.26 million children are blind around the world. Furthermore, 19 million children are visually impaired, including 12 million with uncorrected refractive error—they just need spectacles.^1^ This issue of Community Eye Health Journal - South Asia edition brings you several initiatives to improve children's eye health through school based interventions that have proven to be successful. The WHO programme on School and Youth Health notes that, “An effective school health programme can be one of the most cost effective investments a nation can make to simultaneously improve education and health.”^2^ Integrating eye health into school health programmes can provide comprehensive eye health services to millions of children all over the world. A successful school eye health programme needs to include eye health education, early detection, referral and treatment.^3^ Preventive interventions can address conditions such as conjunctivitis and eye injuries among children. With early detection, conditions like cataract and uncorrected refractive error can be treated on time, leading to improved quality of life. With teachers working closely with children on a day-to-day basis, training and integrating teachers into school eye health programmes can also help identify children with low vision.

This issue begins with an overview that looks at a range of school eye health interventions in South Asia. These interventions highlight the importance of including eye health in school health programmes. Successful eye health programmes in South Asia illustrate experiences of implementing school eye health initiatives and attempt to go beyond screening for refractive errors. In Pakistan, a school eye health programme involved engaging multiple stakeholders, leading to improved access to eye health services for children in rural communities.

An eye health programme in Nepal used a holistic approach involving eye screening, health education and promoting inclusive education in schools. This approach promoted inclusive education for children with disabilities through advocacy for accessible ramps, appropriate classroom settings and sensitisation of children, adults and teachers to the needs of visually disabled children.

In Sri Lanka, mandatory periodic school medical inspections for all children and provision of free spectacles proved to be a successful strategy. Training teachers to conduct initial screening of children in Indian schools showed that teachers can become advocates for child eye care in school as well as in their communities.

Other models of eye care for children applied in different parts of South Asia are showing promise in reaching out to school aged children. A hospital-based community eye health programme in India reached out to children below five years and school dropouts. This model of eye care delivery helped in empowering people, including children, living in the service area of the hospital leading to an improvement in eye health-seeking behaviour and delivery of quality eye care services. In Uttar Pradesh, the most populous state in India, a school eye health programme used a clustered approach to reach out to a large population.

Uncorrected refractive errors, headache and asthenopia, strabismus and amblyopia, developmental cataract, inherited retinal dystrophies and globe anomalies and ocular allergies are some of the ocular problems among school aged children in the region. A brief summary of such conditions, diagnosis and methods of treatment may help in early screening and diagnosis of common eye health problems.

**Figure:**
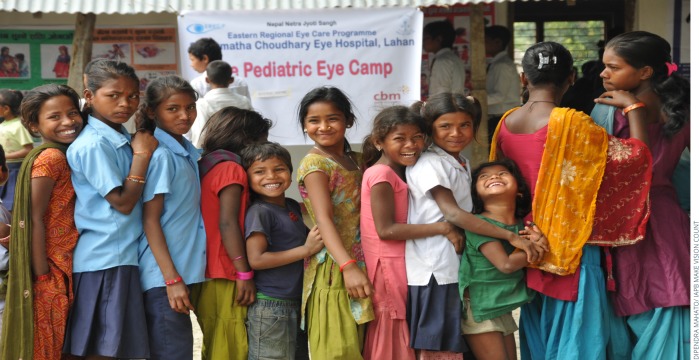
Good vision is vital for a child's growth. INDIA

Through cost effective measures many eye conditions in children are avoidable. A lot can be achieved through school health programmes by including health education, which promotes healthy behaviour and leads to early detection and referral of children with eye problems. Engaging various stakeholders such as ministries of health, NGOs active in education and communities will make a school eye health programme sustainable. With this issue we hope to promote the inclusion of eye health in all school health programmes in the South Asia region.
